# Out of Pocket Expenditure on Health Service Delivery at a Tertiary Care Women’s Hospital: A Descriptive Cross-sectional Study

**DOI:** 10.31729/jnma.5433

**Published:** 2020-12-31

**Authors:** Puja Gartaula, Shristi Neupane, Dip Narayan Thakur, Raj Kumar Sangroula

**Affiliations:** 1 Department of Public Health, Little Buddha College of Health Sciences, New Baneshwar, Kathmandu, Nepal; 2 Nepal Public Health Research and Development Center, New Baneshwar, Kathmandu, Nepal

**Keywords:** *expenditures, direct*, *expenditures, health*, *expenditures, indirect*, *institutional delivery*

## Abstract

**Introduction:**

Institutional delivery in Nepal is increasing in the past decades and has been the priority program of the government of Nepal. However, due to the hidden costs related to institutional deliveries, the financial burden remains unacceptably high for poor households. The study aimed to find out the major out of pocket expenditure on health service delivery at a tertiary care hospital in Kathmandu, Nepal.

**Methods:**

A descriptive cross-sectional study was carried out at a tertiary care hospital from December 2018 to May 2019. Ethical approval was taken from Nepal Health Research Council (ref. no. 2087) and permission was taken from the hospital. Informed consent was taken from the participants. Convenient sampling was done. A semi-structured questionnaire was used as a tool for the interview. Data was entered into Epidata and analyzed using the Statistical Package of the Social Sciences version 23. Descriptive analysis was done using mean, median, standard deviation, interquartile range, frequency, and percentage.

**Results:**

The median out of pocket expenditure of the participants to maternal delivery was NRs. 11720 (7610-20263). The median expenditure was found highest for food and drinking NRs. 2500 (1500-5550) and transportation NRs. 2150 (1400-4543) respectively.

**Conclusions:**

Indirect expenditures were found to be higher than the direct medical expenditures. Accessibility of the birthing centers and health insurance may reduce the costs related to maternal deliveries.

## INTRODUCTION

The reduction in maternal and child mortality has accelerated in a few decades but the scenario of developing countries is different.^1,2^ The reasons behind this is the lack of access to health services, especially among the poor, where healthcare access often imposes a substantial economic burden.^3^ The constitution of Nepal addresses health as a fundamental right and provides a platform for free basic health care services free of cost.^4^

To expand access to safe deliveries, Nepal has initiated the Safe Delivery Incentive Programme (SDIP) in 2006 focusing on reducing the high costs related to accessing healthcare at childbirth.^5^ Despite free delivery and incentive, hidden costs continue to exist.^6^ Different studies conducted in developing countries have shown that the cost of care during childbirth is a major factor for the utilization of maternal health services.^7^ It is important to assess maternal satisfaction with care in order to make it more responsive and culturally acceptable, ultimately leading to enhanced utilization and improved outcomes. At a time when global efforts to reduce maternal mortality have been stepped up, maternal satisfaction and its determinants also need to be addressed by developing country governments. This review seeks to identify determinants of women's satisfaction with maternity care in developing countries. Methods: The review followed the methodology of systematic reviews. Public health and social science databases were searched. English articles covering antenatal, intrapartum or postpartum care, for either home or institutional deliveries, reporting maternal satisfaction from developing countries (World Bank list).

This study aims to find out the major out of pocket expenditure on health service delivery at Paropkar Maternity and Women's Hospital, Teku, Kathmandu.

## METHODS

A descriptive cross-sectional study was conducted to assess the out of pocket expenditure to maternal deliveries at the Paropkar Maternity and Women's Hospital, Teku from December 2018 to May 2019. Ethical approval was taken from Nepal Health Research Council (Reference no: 2087) and permission was taken from Paropkar Maternity and Women's hospital. Written informed consent was taken from the participants. The caretakers (husbands) of the women who recently delivered babies in Paropkar Maternity and Women's hospital were included in the study. The sample size was calculated by the following formula:

$$\begin{array}{ll} \text{n} & \text{= }{\text{Z}}^{\text{2}}\times \text{p}\times \text{(1}-\text{p)}/{\text{e}}^{\text{2}}\\ & \text{= }{\left(1.96\right)}^{\text{2}}\times (0.6)\times (0.4)/{(\text{0.08)}}^{\text{2}}\\ & \text{= }144 \end{array}$$

Where,

n = sample size
Z = 1.96 at 95% Confidence Interval (CI)
p = prevalence of out of pocket expenditure, 60%^8^
e = margin of error

Including a non-response of 15%, the final sample size was 165. The convenient sampling technique was used to select the participants for the study.

The variables under study were socio-demographic and accessibility variables like age, sex, religion, ethnicity, education, occupation, age at first pregnancy, number of children, mode of delivery, and the time taken to reach a health facility. Cost related variables included (i) Direct medical costs: medicines and supplies, laboratory and diagnostic services, blood transfusion (expenditure for arranging blood), doctors fee if applicable; (ii) Indirect medical expenditures like transportation cost for seeking services, expenses on food during the stay in the facility, loss of wages and other category includes all the expenditure that the caregiver could not classify under the specific categories.

Pretested semi-structured Questionnaire was used as a tool for interviews. Face to face interview was used as a technique to find out the major out of pocket expenditure to maternal deliveries. Completeness and consistency of the filled questionnaires were ensured on the same day of the data collection.

Collected data were edited manually to check completeness and accuracy and entered in Epi data. Data analysis was done in IBM Statistical Package for Social Science (SPSS) 23. Descriptive analysis was done using mean, median, frequency, percentage, interquartile range, and standard deviation.

## RESULTS

In the study, the median cost for medicine was NRs. 1700 (1300-4150) and for blood transfusion was NRs. 535 (0 - 567). The median cost for transportation, food, and other (communication, laundry, etc.) was NRs. 2150 (1400-4543), Nrs. 2500 (1500-5550), and Nrs. 500 (350-850) respectively. The median loss of wages of the husband was NRs. 2000 (1600-2666). The total median out-of-pocket health expenditure was NRs. 11720 (7610 - 20263) (Table 1).

**Table 1:** Out of pocket health expenditures of the participants.

Health Expenditure	Mean±SD (NRs.)	Median (IQR) (NRs.)
Direct Expenditure		
Medicines	3684±4891	1700 (1300-4150)
Blood transfusion	502±620	535 (0-567)
Total Direct Expenditure	4186±5250	2035 (1500-4920)
Indirect Expenditure		
Transportation	4137±5044	2150 (1400-4543)
Food	4698±5926	2500 (1500-5550)
Others	642±387	500 (350-850)
Loss of wages of attendants (husbands)	2534±2354	2000 (1600-2666)
Loss of wages of recently delivered women (n= 28)	218±495	-
Total Indirect Expenditure	12230±9986	8750 (5650-14600)
Total Expenditure	16418±13508	11720 (7610-20263)

The age at marriage for 95 (57.6%) respondents was below 20 years of age with a median of 20 years. Similarly, 100 (60.6%) women were pregnant at the age of 20 years or more with a median of 22 years. One hundred and forty-eight (89.7%) respondents had two children or less. Regarding the mode of recent delivery, 108 (65.5%) had a normal mode of delivery and 138 (83.6%) stayed at the hospital for 5 or fewer days. It took less than one hour to reach the hospital for 123 (74.5%) respondents (Table 2).

**Table 2:** Maternal health-related characteristics of the participants.

Characteristics	Frequency n (%)
Age at marriage	
Less than 20	95 (57.6)
More than 20	70 (42.4)
Age at first pregnancy	
Less than 20	65 (39.4)
More than 20	100 (60.6)
Total no. of childbirth	
Less than 2	148 (89.7)
More than 2	17 (10.3)
Mode of delivery	
Normal delivery	108 (65.5)
C- Section	57 (34.5)
Length of stay	
≤ 5 days	138 (83.6)
> 5 days	27 (16.4)
Distance	
Less than 1 hour	123 (74.5)
More than 1 hour	42 (25.5)

One hundred and fifty-five (93.9%) families had a male head and 125 (75.8%) respondents were Hindu. Regarding the ethnicity of the respondents, 87 (52.7%) were Janajati, 62 (37.6%) were Brahmin/Chhetri and 16 (9.7%) were Dalit/Madhesi. For 57 (34.5%) respondents, their husbands' occupation was agriculture followed by labor for 48 (29.1%). Whereas, most of the women were housewives, i.e. 137 (83.0%). Seventy-six (46.1%) participants belonged to a nuclear family, and 48 (29.1%) belonged to a joint family. One hundred and forty-seven (89.1%) delivered women were literate and 51 (33.7%) had some secondary level of education. One hundred and nineteen (72.1%) had a monthly income less than NRs. 25000 and the median family income was NRs. 20000. One hundred and three (63.0%) had family members less than five (Table 3).

**Table 3:** Socio-demographic characteristics of the participants.

Characteristics	Frequency n (%)
Head of Family	
Male	155 (93.9)
Female	10 (6.1)
Religion of Family	
Hindu	125 (75.8)
Buddhist	31 (18.8)
Christian	9 (5.5)
Ethnicity of Family	
Brahmin/Chettri	62 (37.6)
Janjati	87 (52.7)
Dalit/ Madhesi	16 (9.7)
Father’s Education	
Literate	151 (91.5)
Illiterate	14 (8.5)
Level of Education of Father (n= 151)	
Primary	20 (13.25)
Lower secondary	22 (14.57)
Secondary	51 (33.77)
Higher Secondary and more	58 (38.41)
Mother’s Education	
Literate	147 (89.1)
Illiterate	18 (10.9)
Level of Education of Mother (n= 147)	
Primary	29 (19.7)
Lower secondary	79 (53.7)
Secondary	26 (17.7)
Higher secondary or more	13 (8.8)
Occupation of Husband	
Agriculture	57 (34.5)
Service	28 (17.0)
Labor	48 (29.1)
Foreign Employment	13 (7.9)
Business	19 (11.5)
Occupation of Mother	
Housekeeping	137 (83.0)
Service/Labor	41 (17)
Types of Family	
Nuclear Family	76 (46.1)
Joint Family	48 (29.1)
Extended Family	41 (24.8)
Income of Family	
Less than 25000	119 (72.1)
More than 25000	46 (27.9)

## DISCUSSION

Out-of-pocket expenditure in health may harm the household economy of people, especially in developing countries.^9^ Nepal still has a high proportion of out-of-pocket health payment and a limited risk- pooling mechanism. Out-of-pocket payment for the healthcare services could result in catastrophic health expenditure (CHE). This study was conducted to identify out of pocket expenditure in delivery care at Paropakar Hospital, Kathmandu.

In Nepal, out of pocket expenditure has increased by more than 30% during the last 15 years, with the share of OOPS in consumption expenditure increasing from 3.4% in 1995-1996 to 4.5% in 2010-2011.^10^ Many people in Nepal are not able to afford two-way transportation costs despite transport incentives provided by the government for institutional delivery. This study showed median transportation cost was NRs. 2150. The median cost for food and drinking was highest among the costs with NRs. 2500. In western Nepal, about 53.07% of total expenditure was food and drinking and contributed the highest among all expenditure.^11^ A study conducted in Nigeria showed that expenditure after child care was higher for food.^12^ Similarly, a major share in out-of-pocket expenditure in India was Tips and the median value of OOPE was 320 INRs.^3^ A study conducted in tertiary hospitals of Bangladesh showed that costs for medicine, transportation, and food were the first, second, and third major expenditures respectively.^13^ The findings from this study suggest that hospital attendees should also be focused by the government as expenditure is higher for food and drinking. In this study, the median loss of wages of the attendants was NRs. 2000 and the median loss of wages for the recently delivered women were negligible as many were housewives but a study conducted in the Western part of Nepal had a higher loss of wages of both the husbands and wives.^14^ This may be due to the different length of stay at the hospital. The study showed median total expenditure on delivery was NRs. 11720 which is more than 50% of the total monthly income of the family. While in India the median expenditure in delivery was only INRs. 700.3 But the median expenditure was much lower than a study conducted in tertiary hospitals of Western Nepal.^11^ The different findings in total expenditure may be due to less cesarean section which decreases the length of stay and distance of hospital from home.

## CONCLUSIONS

In this study, the expenditure on basic needs like food and drinking was higher among other expenditures. Expenditure on transportation contributed secondly to the total delivery related expenditure. These costs may be barriers to hospital delivery for families with the poor socio-economic condition. Increasing coverage of health insurance and accessibility of birthing centers may reduce the costs of pregnant women attending healthcare for delivery.
